# Nationwide Routine Childhood Vaccination Coverage During the COVID-19 Pandemic in Jordan: Current Situation, Reasons, and Predictors of Vaccination

**DOI:** 10.1155/2022/7918604

**Published:** 2022-03-24

**Authors:** Eman Y. Abu-rish, Yasser Bustanji, Kamel Abusal

**Affiliations:** ^1^Department of Biopharmaceutics and Clinical Pharmacy, School of Pharmacy, The University of Jordan, Amman, Jordan; ^2^Department of Basic Medical Sciences, College of Medicine, University of Sharjah, Sharjah 27272, UAE; ^3^Hamdi Mango Center for Scientific Research, The University of Jordan, Amman, Jordan; ^4^Department of Vaccination, Communicable Disease Directorate, Ministry of Health, Amman, Jordan

## Abstract

**Background:**

The healthcare system in Jordan faced substantial burden during the 2020 COVID-19 pandemic including disruption of routine childhood vaccination services.

**Aims:**

We sought, for the first time, to describe the impact of the 2020 pandemic on vaccination coverage of Jordanian children in Jordan and to identify the key contributing factors.

**Methods:**

Nationwide vaccination rates were retrieved from the electronic records at the Ministry of Health (2018–2020) enrolling crude births of 220,057 Jordanian children during 2020. Records of doses administered were compared for each month of 2020 with the baseline of 2018–2019. A cross-sectional survey (March–August 2021) was also conducted enrolling a convenient sample of adults aged ≥18 who were Jordanian caregivers for vaccine-eligible children (0–23 months) between 1 January 2020 and the date of the interview. The survey aimed to address caregivers' adherence to routine vaccination during 2020–2021 and to describe the determinants of the current and future adherence to vaccination where multiple logistic regression model was utilized.

**Results:**

The electronic records revealed a significant decline in vaccination coverage during 2020. The greatest decline was observed during the lockdown period from 21 March 2020 to 21 April 2020 (32.4%–46.8%) followed by the decline observed by the entry of the first wave during September-October 2020 (18.4%–22.8%). A drop of 14–16% was observed for the vaccines recommended under the age of 12 months and of 6–7% for those recommended in 1-2-year-old children. The yearly coverage rates for measles-1 (at 9 months), 2 (at 12 months as part of measles-mumps-rubella (MMR) vaccine), and 3 (at 18 months as part of MMR) were 76%, 90%, and 87%, respectively, and for hexavalent-1, 2, and 3 were 78%, 78%, and 77%, respectively. The results of the survey revealed that the main reason for vaccination delay for at least 1 month from the recommended administration time was the lockdown, followed by child illness and smart lockdowns (regional lockdown/health center closure). Vaccination delay was less likely to be observed in children aged ≥12 months (*P* value < 0.001; OR: 0.18; CI: 0.11–0.29) or children with chronic diseases (*P* value < 0.05; OR: 0.5; CI: 0.33–0.88).

**Conclusion:**

The current study demonstrates a decline in vaccination coverage of Jordanian children during the 2020 COVID-19 pandemic. It is important to formulate future strategies to promote catch-up vaccination and to avoid future backsliding of vaccination rates during further waves of the COVID-19 pandemic or other pandemics. These include improving health services, allaying caregivers' concerns about contracting COVID-19, and arranging vaccination campaigns outside health centers.

## 1. Introduction

Since the onset of the coronavirus disease-19 (COVID-19) pandemic, the disruption of global public health services has commenced. This disruption has resulted from the implementation of several COVID-19 containment measures and from the COVID-19-related burden on health systems. Interruption of childhood vaccination services was one of the major pandemic fallouts, where 23 million children worldwide have missed out routine immunization during 2020 as reported by the World Health Organization (WHO) and the United Nations International Children's Emergency Fund (UNICEF) in July 2021 [1]. Several early reports described a decline in vaccination coverage during the pandemic in Pakistan [2], England [3], USA [4], Saudi Arabia [5], Canada [6], and many other countries [7, 8]. Similar, or even worse, situation is expected to be observed in the limited-resources Eastern Mediterranean country of Jordan that is located in an area of conflicts and is a host for millions of refugees, which probably would amplify the consequences of the pandemic.

Jordan was substantially hit by two waves of COVID-19 pandemic. Although the first case of COVID-19 was recorded on 2 March 2020, it was not before September 2020 that the number of COVID-19 cases dramatically increased, alarming the entry of the first wave of the pandemic that peaked in November 2020 [9]. The second wave began in February 2021, peaked in March 2021, and lasted near the end of May 2021 [10]. Since then, the epidemiological situation in Jordan is considered stable with non-alarming fluctuations in the numbers of COVID-19 cases [10].

The low incidence of COVID-19 and the flat curve characteristic of the early pandemic stage (March–August 2020) are attributed to the enforcement of stringent containment measures, including lockdown and curfews [9]. Lockdown was imposed in Jordan on 21 March 2020 and was extended several times until 30 May 2020. During this period, lockdown was gradually relaxed to allow people walking for grocery shopping. Only exempted cars were initially allowed to move for essential services such as healthcare services. On 21 April 2020, health centers were reopened for child immunization purposes. However, car movement was not allowed until 29 April 2020 when alternate even-odd car license plate policy was applied along with reopening of the economic field. After 30 May 2020, smart lockdown was implemented, involving regional lockdown in areas with rising numbers of COVID-19 cases or closure of certain sectors [11].

The immunization program in Jordan (Table 1) starts by the administration of *tuberculosis* vaccine (Bacillus Calmette–Guérin [BCG] vaccine) the soonest after delivery. Three doses of each of rotavirus diarrhea vaccines and hexavalent vaccines (diphtheria, pertussis, tetanus (DPT); *Haemophilus influenzae* type b (Hib); hepatitis B; and inactivated polio vaccine (IPV) are administered at the age of two, three, and four months. Three doses of oral polio virus (OPV) vaccine are recommended at the age of three, four, and nine months besides a booster dose at the age of 18 months. The first dose of measles vaccine is recommended at the age of 9 months; the second and the third doses are given at the age of 12 and 18 months as part of the trivalent viral vaccine (measles, mumps, and rubella [MMR]). A booster dose of the trivalent bacterial vaccine (DPT) is also given the age of 18 months. As of July 2020, hepatitis A vaccine was introduced into the national immunization program in Jordan and is given at a two-dose schedule at the age of 12 and 18 months [12].

Adherence to routine childhood vaccination is crucial to avoid the outbreak of vaccine-preventable diseases which becomes a global concern at times of pandemics such as the COVID-19 pandemic [13]. Therefore, it is essential to monitor vaccination coverage and to develop strategies to improve this coverage during pandemics. Hence, in this report, we describe for the first time the impact of the COVID-19 pandemic on vaccination coverage in Jordan during 2020 using the electronic records at the Ministry of Health (MOH). We also aimed to clarify the determinants of vaccination delay during 2020–2021 years of the COVID-19 pandemic and the predictors of adherence to future vaccination through a survey-based cross-sectional study. The findings of this study will provide implications for the formulation of future preparedness plans to ensure the adherence to routine childhood vaccination during pandemics in Jordan.

## 2. Methods

### 2.1. Data Source and Study Procedure

Jordan is a home for about 10,993,553 people [14] with 12 governorates including the capital Amman, Balqa, Madaba, Zarqa, Ajlun, Jerash, Mafraq, Aqaba, Karak, Ma'an, and Tafilah. Routine vaccination services, for 0–23-month-old children, are provided through public health centers (549 centers as of 2019) [15], United Nations Relief and Works Agency for Palestine Refugees (UNRWA) primary health facilities (25 centers) [16], and private clinics.

**Figure 1:**
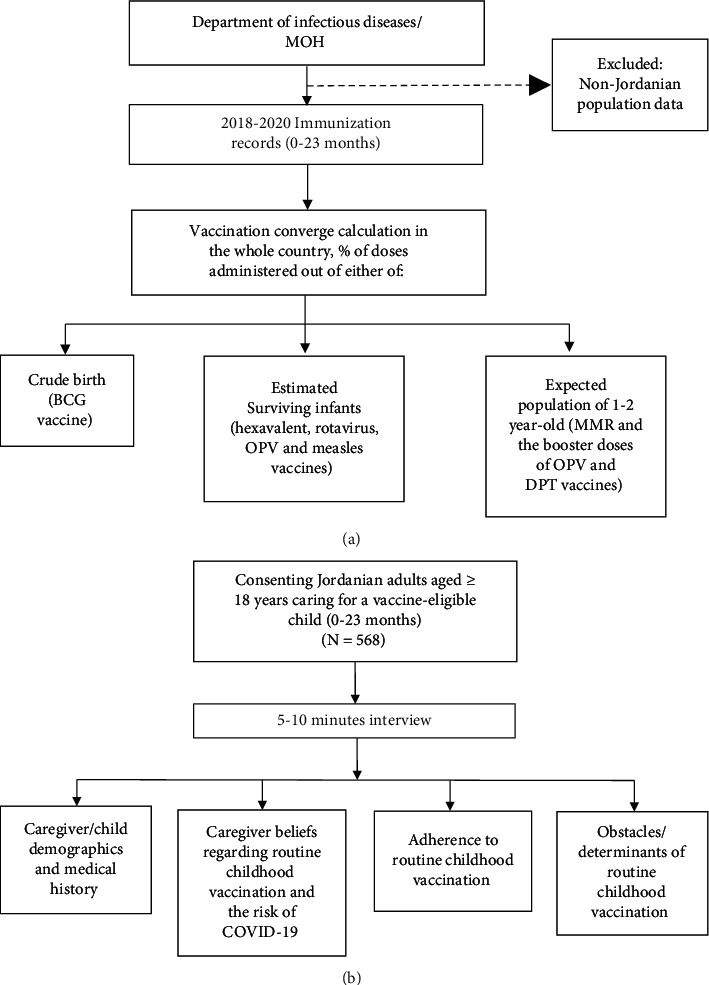
Flowchart of the study design. (a) Nationwide 2018–2020 childhood vaccination coverage as retrieved from the immunization records at the Ministry of Health. (b) Survey-based study of routine childhood vaccination coverage during COVID-19 pandemic 2020-2021. BCG: Bacillus Calmette–Guérin; DPT: diphtheria, pertussis, and tetanus; MMR: measles, mumps, and rubella; MOH: Ministry of Health; OPV: oral polio vaccine.

In order to assess the impact of the pandemic on childhood vaccination in Jordan, data were obtained from two sources: the electronic records of vaccination of children with Jordanian nationality at the MOH and a survey-based cross-sectional study (Figure 1).

Routine childhood vaccination records (0–23 months) for Jordanian population, through the years 2018–2020, were obtained from the Vaccination Department, Communicable Disease Directorate, MOH. Data retrieved from 2018-2019 records were used as a baseline against which data during 2020 was compared. Institutional review board (IRB) approval of the study protocol was obtained by the MOH (Moh/REC/2021/024). The target population in this study was limited to those of Jordanian nationality (estimated population of 7,287,488 and estimated cohort of children <2 years of 407,733) [17]. This is because an estimation of the vaccination coverage for non-Jordanians could not be determined due to the lack of accurate statistical estimation of crude births and surviving infants for this category (Figure 1(a)).

As the records of the MOH do not include caregiver/child demographics and medical history, a population-based cross-sectional survey was also conducted between March and August 2021 in order to gather such information and to reveal the factors associated with adherence to or hesitancy about routine childhood vaccination during the pandemic (Figure 1(b)). The survey included a target sample of 400 adults aged ≥18 who were Jordanian parents/caregivers for vaccine-eligible children (0–23 months) between 1 January 2020 and the date of the interview. The children included should have had at least one scheduled vaccine between 1 January 2020 and the end of the study interval. In case of more than one eligible child for a caregiver, one of them was randomly included in the study. The sample size was estimated based on the following equation: *N* =  *PQ*(*Z*_*α*_+*Z*_*β*_)^2^/*d*^2^, where N is the sample size; *Zα*: type one error = 1.96 when *a* = 5%; *Zβ*: type two error = 1.28 when *ß* = 10%; *Q* = 1-P: expected non-prevalence; *P* is the proportion of the population possessing the characteristics of interest (based on an average of 90% of all-vaccine coverage among 0–23-month-old Jordanian children during 2019 as provided by the MOH); *d* is one-half of the desired interval of confidence; and in this study *d* = 5%. To account for possible missing data, the sample size was inflated to around 600 participants.

Survey data was collected by a research assistant though a 5–10-minute interview with Jordanian participants after obtaining oral consent. Participants were attending the outpatient clinics at Jordan University Hospital (JUH) or Al-Esraa Hospital for medical appointments for themselves or for their children. Both hospitals are located in the capital Amman. JUH was chosen for this study as it is a public hospital that receives patients from almost all governorates in Jordan with 668,870 patients attending the hospital during 2019. AL-Esraa Hospital was chosen as a convenient private hospital. Approval was obtained by the institutional review board (IRB) at JUH (10/2021/4339).

The questionnaire—presented as a Google form—was built after reviewing related literature [5, 18, 19]. It was face-validated by several colleagues in the field of clinical pharmacy and was then piloted to a sample of 20 participants (5% of the target sample size). The data obtained from piloting were not included in the final analysis. The questionnaire was structured into several sections including caregiver/child demographics (age, gender, health status, and presence of child's chronic illness), caregiver's beliefs regarding routine childhood vaccination and the risk of acquiring COVID-19, child vaccination status and practices of adherence to childhood vaccination during 2020-2021, and obstacles to and predictors of the current and future adherence to childhood vaccination. Caregiver-reported child vaccination status was recorded or was verified from the vaccination card if available with the caregiver at the time of the interview.

### 2.2. Statistical Analysis

Statistical analysis was carried out using SPSS version 25.0 (SPSS Inc., Chicago, IL).

The yearly vaccine coverage rate in the whole country was calculated as a percent of the yearly count of vaccine doses administered out of the corresponding yearly crude birth rate for BCG vaccine, the yearly surviving infant population for the vaccines administered during the first year of life, or the expected population of 1-2-year-old for the rest of the vaccines. The monthly vaccine coverage was calculated using the monthly counts of the vaccine doses administered as percentages of the monthly crude births, the monthly surviving infant population, or the monthly expected population of 1-2-year-old for the aforementioned designated vaccines. The estimated monthly population was calculated by dividing the estimated yearly population by 12.

Categorical variables were presented as percentages with frequencies, whereas continuous variables were presented as median with interquartile range (IQR). The primary outcome measure of this study was “vaccination delay” which is defined in the current study as a vaccination that was administered after one month or more of the recommended vaccination age besides those vaccinations that were delayed and had not yet been administered by the time of the interview. Kruskal–Wallis test was employed to find out between-group differences. Bivariate analyses using Pearson's chi-square or Eta-squared tests were conducted as appropriate to find out associations between the outcome measure of “vaccination delay” and the potential predictors of this outcome. A multiple logistic regression model was then developed including those covariates with significant association with the outcome measure. The “no” answer for vaccination delay was scored 0, and the “yes” answer was scored 1. All hypothesis tests were two-sided. A *P* value of <0.05 was considered to be statistically significant. Graphs were constructed using Prism 9.0.0 software (GraphPad, USA).

## 3. Results

### 3.1. Nationwide Routine Childhood Vaccination Coverage during 2018–2020

#### 3.1.1. The Drop of Vaccination Coverage during the 2020 COVID-19 Pandemic

During 2020, vaccination coverage per type of vaccine was significantly reduced as compared to the average coverage during 2018 and 2019. This drop ranged between 6% and 16% (Table 2). Regardless of the type of the vaccine, the total number of vaccine doses administered and the average percentage of vaccine coverage were significantly reduced in 2020 (*n* = 2,497,794; 79.6%) as compared to either 2019 (*n* = 2,702,482; 90.7%; *P* value = 0.001) or 2018 (*n* = 2,773,570; 93.5%; *P* value <0.001) (Table 2). The annual coverage of hepatitis A vaccine was not included in the analysis as it was introduced in July 2020 for the first dose and in January 2021 for the second dose.

#### 3.1.2. The Monthly Change in Vaccination Coverage during the 2020 COVID-19 Pandemic as Compared to 2018 and 2019

**Figure 2:**
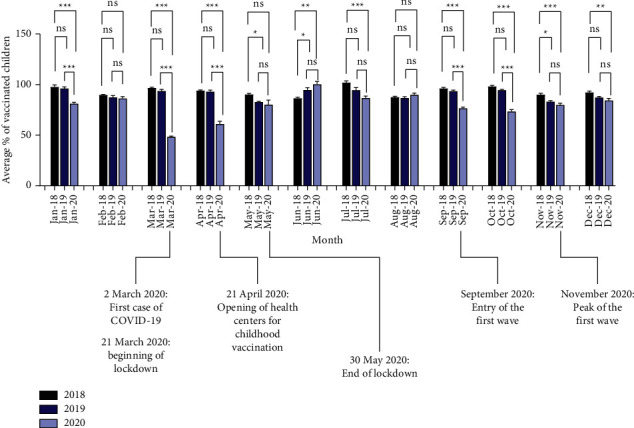
Monthly vaccination coverage during 2018–2020. Important COVID-19 pandemic-related dates are presented. Data represent mean ± SEM; *n* = 15 (number of vaccines); Kruskal–Wallis test;^*∗*^*P* value <0.05, ^*∗∗*^*P* value <0.01, ^*∗∗∗*^*P* value <0.001).

The analysis of the monthly vaccination coverage during 2020 demonstrated a significant reduction in coverage during January 2020 as compared to January in either of the previous two years (Figure 2). The drop became significantly obvious during March and April 2020 with vaccination coverage of 48.3% ± 0.91 and 61.1% ± 2.75, respectively, as compared to the coverage during the corresponding months in 2018 (96.5% ± 0.88 and 94.0% ± 0.76, respectively) or 2019 (93.7% ± 1.47 and 92.9% ± 1.61, respectively). Coverage was increased during May 2020 to levels similar to those of the previous two years and became even higher than the previous two years during June 2020. Coverage was reduced again during September and October 2020 with the entry of the first wave (76.5 ± 1.4 and 73.47 ± 1.87, respectively) as compared to the corresponding months during 2018 (96.1 ± 1.23 and 98.0 ± 1.22, respectively) or 2019 (93.6 ± 0.96 and 94.5 ± 0.77, respectively). The decline, though less prominent, continued until December 2020.

### 3.2. Survey-Based Analysis of Routine Childhood Vaccination Coverage during the COVID-19 Pandemic (2020-2021)

#### 3.2.1. General Characteristics of Participants

Of the 588 adults approached, 568 consented to participate with 96.6% response rate.

The median age of the caregivers was 30 ± 8 (years ± IQR) (18–59 years) and the median age of children was 12 ± 14 (months ± IQR) (1–39 months). The sociodemographic characteristics of the caregivers and their children are presented in Table 3.

Participants included in this study were mainly from Amman governorate (*n* = 215, 62.5%), followed by Balqa (*n* = 62, 18%), Zarqa (*n* = 33, 9.6%), Madaba (*n* = 11, 3.2%), Irbid (*n* = 7, 2%), and Jerash (*n* = 4, 1.1%), and 3.6% (*n* = 12) were from the rest of the governorates (excluding Ajlun, *n* = 0).

#### 3.2.2. Caregivers' Beliefs about Routine Childhood Vaccination and Adherence to Vaccination during the COVID-19 Pandemic

Assessment of caregivers' beliefs (*N* = 568) about childhood vaccination and the adherence to vaccination schedule during the COVID-19 pandemic revealed that most of them believed in the importance of childhood vaccination for child health (90.5%) and in the importance of adherence to child vaccination schedule (93.1%). Being at risk of acquiring COVID-19 when visiting health centers for child vaccination was believed by 71.5% of them (Table 4).

#### 3.2.3. Practice and Determinants of Current and Future Adherence to Childhood Vaccination during the COVID-19 Pandemic

Of the 568 caregiver interviewed, 25% (*n* = 142) delayed at least one vaccine for a child since 1 January 2020 until the time of the interview. Fifty-seven percent (*n* = 81) of those who had delayed child's vaccines admitted that the delay was related to the emergence of COVID-19. The most frequent duration of the delay was one month (*n* = 58, 40.8%) followed by 2 months (*n* = 38, 26.8%) (Table 5). The most frequently delayed vaccine was the eighteenth-month vaccine (*n* = 74, 33.1%), and around 20% of the delays were encountered for the first-month vaccine or the vaccines recommended between third and twelfth months, while 13.4% (*n* = 19) of the delays were reported for the second-month vaccine (Table 5). It is worth mentioning that of the 142 participants who delayed a child's vaccine, 25.4% (*n* = 36) delayed 2 or more vaccines while 74.6% (*n* = 106) delayed 1 vaccine. Consequently, the total number of the delayed doses since 1 January 2020 until the time of the interview was 203 doses (Table 5). Notably, a total of 129 doses of the vaccines recommended for children younger than 12 months were delayed as compared to 74 doses of the vaccines recommended for children at the age of 12 months and more.

The most frequently reported time interval during which the vaccines were delayed was the interval after 30 May 2020 until the end of the study interval (*n* = 74, 52.5%) followed by the interval of the lockdown (21 March 2020 to 21 April 2020) (*n* = 60, 42.3%) (Table 5).

The main reported reasons for the delay were the lockdown (*n* = 60, 42.3%) and child illness at the vaccine due date (*n* = 45, 31.7%) followed by smart lockdowns (regional lockdown/health center closure due to COVID-19) (*n* = 11, 7.7%) while other reasons were less frequently reported (Table 5).

Upon questioning participants (*N* = 568) about the factors that would encourage them to adhere to routine childhood vaccination in the future, the most important factor was the organization of awareness campaigns regarding the importance of adherence to routine childhood vaccination during the COVID-19 pandemic (*n* = 516, 90.8%), followed by establishing child vaccination campaigns outside health centers to reduce the risk of acquiring COVID-19 (*n* = 431, 75.9%) and the supply of childhood vaccination at home during the pandemic (*n* = 348, 61.3%).

The most important sources of medical information regarding routine childhood vaccination (*N* = 568) were health centers (*n* = 232, 40.8%), Internet (*n* = 166, 29.2%), physician clinics (*n* = 84, 14.8%), UNRWA (*n* = 25, 4.4%), and others (*n* = 61, 10.7%). The source of medical information was not associated with the outcome of “vaccination delay” (*P* value >0.05).

#### 3.2.4. Multiple Logistic Regression Model for the Predictors Associated with Vaccination Delay

As per the results of the bivariate analyses, the covariates which were found to be significantly associated with the outcome measure of “vaccination delay,” as shown in Tables 2 and 3, were entered into a multiple logistic regression model (Table 6). Accordingly, caregivers who had a child with a chronic disease (*P* value <0.05; OR: 0.53; 95% CI: 0.33–0.88) or at the age of 12 months or more (*P* value <0.001; OR: 0.18; 95% CI: 0.11–0.29) were significantly less likely to delay a child's vaccine. However, caregivers who believed in the importance of adherence to child vaccination schedule were 4-fold more likely to delay the vaccination.

## 4. Discussion

The data retrieved from the electronic records of MOH and the findings of the cross-sectional study presented in this report show that, in Jordan, adherence to childhood vaccination programs in 2020 was a challenge with a significant drop in vaccination coverage. This was reflected in the significant overall decline in the electronic record-based vaccination coverage during 2020 by 13.9% and 11.1% as compared to 2018 and 2019, respectively. While the electronic record-based estimates reflect the decline in vaccination uptake during the year of 2020 only, the survey results reflect the decline since the beginning of 2020 up to the end of data collection in August 2021. Hence, a 25% vaccination delay was revealed through the survey results. According to the WHO and other cross-sectional surveys, global reduction in vaccination uptake was observed during the 2020 COVID-19 pandemic [1, 7, 8].

Analysis of coverage per vaccine demonstrated an electronic record-based decline by 14–16% for the vaccine administered under the age of 12 months and by 6–7% for the vaccine administered for the 1-2-year-old children as compared to the average of the previous two years. In line with this, the survey-based data demonstrated that 129 doses of the vaccines recommended for children younger than 12 months were delayed as compared to 74 doses of the vaccines recommended for children at the age of 12 months and more. It is worth mentioning that about a quarter of those caregivers who delayed vaccines had delayed two or more vaccines for a child. This age-related difference in vaccination uptake was reflected in the results of the regression analysis of the predictors of vaccination delay where caregivers of children at the age of 12 months or more were significantly less likely to delay a child's vaccine. This could be attributed to the probably higher caregivers' concerns about exposing their vulnerable newborns and other children under the age of 12 months to the risk of COVID-19. This age-related difference in vaccination coverage during the pandemic was also documented in Colombia where the highest decline in vaccination coverage during March-October 2020 was observed in children under the age of 12 months, in particular those living in rural areas [20]. Similarly, in Michigan, around 17% reduction in the percentage of vaccinated children at the age of 5 months was documented in May 2020 as compared to around 10% and 5% reduction at ages of 19 months and 24 months, respectively [4].

During previous health emergencies, such as the Ebola epidemic in West Africa, the disruption in health systems and vaccination services resulted in secondary measles outbreak [21]. During 2020, the electronic records of vaccination coverage in Jordan showed that the total coverage of the first, second, and the third dose of measles vaccine was 76%, 90%, and 87%, respectively, which is generally below the level required to establish herd immunity (89%–94%), hence exposing the community to the risk of measles outbreak [22]. Compared to the situation in other countries, the coverage of the first dose of measles vaccine in Jordan was reduced by 14% (19% as per the survey) while, according to the WHO, no reduction was reported in the higher income countries of England or USA [23]. As compared to other neighboring Middle Eastern countries, 0.5% reduction was documented in the high-income country of Saudi Arabia and 1% reduction in the lower-middle-income country of Egypt [23]. Likewise, the coverage of other critical vaccines such as DPT-3 vaccine, as part of the hexavalent-3 vaccine, was reduced by 16% during 2020 (20.4% as per the survey) while no reduction or only 1% reduction was reported in the UK or the USA, respectively, and 1% reduction was reported in both Saudi Arabia and Egypt [23]. Similar scenarios apply for the coverage of the rest of the vaccines when compared to the aforementioned countries. It is important to notice that these comparisons are based on yearly coverage rates provided to the WHO by the national government in the aforementioned countries. Therefore, disruption occurring during lockdown periods should be taken into account [24] (discussed in the following paragraph). Although Jordan is classified by the World Bank as an upper-middle-income country, the high influx of Syrian refugees, together with the pandemic, has placed the health system in Jordan under much of strains resulting in the observed reduction in vaccination coverage. Therefore, international support might be needed to avoid vaccine-preventable disease outbreak in Jordan.

The monthly analysis of electronic record-based total vaccination coverage during the year of 2020 as compared to baseline coverage demonstrated that the greatest reduction was observed during March-April 2020 (46.8% and 32.4% reduction as compared to the average coverage of the corresponding months in 2018 and 2019, respectively). This could be attributed to the growing fear of acquiring COVID-19 after recording the first case in Jordan on 2 March 2020 and the disruption resulted from the lockdown which was imposed later that month through April 2020. Coverage was raised during May 2020 after the opening of health centers for child vaccination and the relaxation of lockdown. Coverage became even higher than the previous two year during June 2020 after the end of lockdown on 30 May 2020 when caregivers were urged to vaccinate their children. Nearly stable coverage was observed during July-August 2020 in parallel with the stable epidemiological situation in the country, before significantly declining again during September and October 2020 with the entry of the first wave of the pandemic (18.4% and 22.8% reduction as compared to the average coverage of the corresponding months in 2018 and 2019, respectively). In other countries, previous reports demonstrated a decline in routine childhood vaccination coverage; however, most of these reports addressed this issue during the early phase of the pandemic while in our report we presented a 12-month analysis of vaccination coverage [2–4, 7, 8]. For instance, compared to higher income countries, the decline observed in Jordan during the early phase (March-April 2020) was higher than that observed in Michigan, USA, or in England [3, 4]. However, a higher decline was reported in the province of Sindh, in the low-middle income country of Pakistan, where 52.5% the daily average total number of vaccinations administered was reduced during the lockdown period [2].

The key findings of the cross-sectional survey were the identification of the reasons and the determinants of routine childhood vaccination uptake during the COVID-19 pandemic. Although more than 90% of the caregivers believed in the importance of vaccination for a child's health and the importance of adherence to vaccination schedule during the pandemic, a quarter of them delayed at least a vaccine for a child. This indicates that factors other than wrong beliefs justify the observed vaccination delays, as 57% of those who delayed a vaccine admitted that the delay was related to the emergence of COVID-19 pandemic. Specifically, the main reported reason for the delay was the early-phase lockdown followed by child illness at the vaccine due date. Other COVID-19 related reasons included regional lockdown/health center closure due to the presence of COVID-19 cases. In parallel with our results, previous reports in USA, England, and Saudi Arabia similarly reported that the main barriers against adherence to childhood vaccination during the COVID-19 pandemic were mainly related to the pandemic, such as disruption of vaccine delivery services or the fear of contracting COVID-19 [5, 25–27]. The reasons reported in our study coincide with the intervals during which vaccination was delayed. 42.3% of the delays occurred during the lockdown although it was relatively short. On the other hand, 52.5% of the delays occurred during the interval extending from post-lockdown period through August 2021, during which the first and the second waves of COVID-19 pandemic hit the country and smart lockdowns (including regional lockdown/health center closure) were implemented. Most of the delays (40.8%) were for a duration of 1 month. However, about a quarter of the caregivers delayed vaccines for 2 months and a quarter for 4 months or more. These long durations of vaccination delay would probably be associated with a higher likelihood of disease outbreak.

A recent risk-benefit analysis has shown that the deaths prevented by sustaining routine childhood vaccination in Africa outweigh the risk of COVID-19 deaths associated with the visits to vaccination clinics [28]. Three factors were revealed in this study to enhance adherence to childhood vaccination during the COVID-19 pandemic. These are organizing awareness campaigns regarding the importance of adherence to childhood vaccination during the COVID-19 pandemic, establishing child vaccination campaigns outside health centers to reduce the risk of acquiring COVID-19, and supplying child vaccination at home. Likewise, about one-third of parents preferred the administration of child vaccination at home during the pandemic in Saudi Arabia [5]. It was also previously recommended that child vaccination are arranged outdoors or in alternative settings, other than health centers, to ensure child-wellness visits during the COVID-19 pandemic [4, 7].

The results of the survey-based regression analysis of the factors associated with vaccination delay demonstrated that none of the caregivers' sociodemographic characteristics was associated with the practice of vaccination delay during the pandemic. However, child's characteristics including age of 12 months and more and having chronic diseases were significantly associated to lower likelihood of vaccination delay. As mentioned above, the age-related vaccination delay could be attributed to the probable caregivers' fears of exposing their vulnerable children under the age of 12 months to the risk of contracting COVID-19. In addition, caregivers of children with chronic illness probably have higher concerns and awareness about the optimization of vaccination status of their children in order to improve their quality of life. Children with chronic diseases are at a markedly higher risk of sever infectious diseases and their complications, which necessitates optimization of their vaccination status [29]. Other reports stated that large household and lack of insurance were significantly related to vaccination delay during the pandemic [5]. Prior to the COVID-19 pandemic, several sociodemographic factors were found to influence childhood vaccination rate such as insurance, income, parental education, or cultural beliefs [25]. In Jordan, the main factors associated with adherence to childhood vaccination prior to the pandemic were child's age, birth order, mother's educational level, and mother's and father's employment status [12]. Our results emphasize that the pandemic had a major impact on vaccination uptake in Jordan which masked the usual influence of the sociodemographic factors on routine childhood vaccination uptake as reported before [12]. Of note, in our report, caregivers who believed in the importance of adherence to childhood vaccination schedule were more likely to delay the vaccination. This is not surprising knowing than 93.1% of the study population and 83.8% of those who delayed the vaccine believed in the importance of childhood vaccination. However, this further emphasizes that the COVID-19 pandemic and the mitigation measures implemented under the national defense orders were the leading determinants for vaccination delay which enforced caregivers to delay child vaccination regardless to their beliefs about adherence to child vaccination.

## 5. Limitations

Although the current study has resulted in interesting findings, it has some limitations. This study presents routine childhood vaccination coverage in Jordanian children only, excluding non-Jordanians, despite the fact that about three million refugees reside in Jordan. Future work should address the coverage in non-Jordanians after the determination of accurate statistical estimation of crude births and surviving infants for this category of population which are currently not available. Furthermore, the electronic record-based data provided in the current study was only for the 2020 year of the COVID-19 pandemic, as the electronic records for 2021 have not yet been verified by the MOH at the time of the preparation of this work. Thus, future studies are required to assess the effect of COVID-19 pandemic on routine childhood vaccination during 2021 using the MOH-verified electronic records and to investigate the impact of launching the COVID-19 vaccine in Jordan on routine childhood vaccination coverage during that year.

Considering the cross-sectional study, 62% of the participants were from Amman with fewer percentages from the rest of the governorates. Therefore, the results of the cross-sectional study should be carefully generalized to the other governorates. It is important to notice that this distribution is relatively in parallel with the distribution of children <2 years in Jordan in 2020, where 158,063 of the estimated 407,733 children of this cohort were from Amman. However, other factors might influence the distribution of participants included from other governorates such as the distance between the governorate and JUH in Amman; for example, Balqa is the nearest followed by Zarqa, which could justify the fact that the percent of participants included from Balqa was higher than that from Zarqa although 27,132 of children <2 were from Balqa compared to 60,893 from Zarqa.

Furthermore, a recall bias in relation to vaccination data obtained from the survey study might have occurred. The survey data was collected in March–August 2021, and the participants were questioned regarding vaccination status of their children since 1 January 2020 until the date of the interview in 2021. Some measures were taken to reduce this bias such as asking caregivers to check child vaccination cards; however, the card was not necessarily available at the time of interview. Linking the recall of a child vaccination status to the child age at specific time intervals (such as lockdown) was also implemented.

## 6. Conclusion

A significant overall reduction in childhood vaccination uptake was observed in Jordan during the COVID-19 pandemic. As Jordan is located in an unstable zone and receives high numbers of refugees, international support is required to improve the current strained health system in Jordan and thus sustain childhood vaccination services during the COVID-19 pandemic. Moreover, formulation of future strategies that promote routine childhood vaccination uptake and catch-up vaccination is necessary to avoid backsliding of vaccination rates during further waves of the COVID-19 pandemic or future pandemics. These strategies include allaying the concerns of caregivers about contracting COVID-19 during vaccination visits and arranging vaccination campaigns outside the health centers or even providing them at home.

## Figures and Tables

**Table 1:** National immunization program in Jordan (0–23 months).

Age	Vaccine
Birth (soonest after delivery)	BCG
2 months (61 days)	Hexavalent-1 (DPT, Hib, HBV, IPV); rotavirus-1
3 months (91 days)	Hexavalent-2 (DPT, Hib, HBV, IPV); rotavirus-2; OPV-1
4 months (121 days)	Hexavalent-3 (DPT, Hib, HBV, IPV); rotavirus-3; OPV-2
9 months	Measles; OPV-3
12 months	MMR-1; hepatitis A-1
18 months	MMR-2; DPT-booster; OPV-booster; hepatitis A-2

BCG: Bacillus Calmette–Guérin; DPT: diphtheria, pertussis, and tetanus; HBV: hepatitis B virus; Hib: *Haemophilus influenzae* type b; IPV: inactivated polio vaccine; MMR: measles, mumps, and rubella; OPV: oral polio vaccine.

**Table 2:** Vaccination coverage during COVID-19 pandemic (2020 compared to 2018 and 2019).

	2018	2019	2020	2018, 2019	Avg. 2018, 2019 vs. 2020
	% (*n*)^†^	% (*n*)^†^	% (*n*)^†^	Avg. %	% change
BCG	94 (189942)^‡^	86 (180313)^‡^	76 (167745)^‡^	90	14
Hexavalent-1^§^	95 (189676)^¶^	90 (184122)^¶^	78 (168208)^¶^	93	15
Hexavalent-2^§^	96 (191540)^¶^	90 (183262)^¶^	78 (169043)^¶^	93	15
Hexavalent-3^§^	96 (190112)^¶^	89 (183093)^¶^	77 (166701)^¶^	93	16
Rotavirus-1	94 (187411)^¶^	88 (179284)^¶^	77 (165090)^¶^	91	14
Rotavirus-2	95 (188615)^¶^	88 (179874)^¶^	77 (165231)^¶^	92	15
Rotavirus-3	93 (185071)^¶^	86 (176605)^¶^	75 (161585)^¶^	90	15
OPV-1	96 (191540)^¶^	90 (183262)^¶^	78 (169043)^¶^	93	15
OPV-2	96 (190112)^¶^	89 (183093)^¶^	77 (166701)^¶^	93	16
OPV-3	92 (182975)^¶^	87 (178913)^¶^	76 (164475)^¶^	90	14
OPV-booster	89 (172226)^‖^	97 (176845)^‖^	86 (164611)^‖^	93	7
Measles	92 (182975)^¶^	87 (178913)^¶^	76 (164872)^¶^	90	14
MMR-1	96 (186923)^‖^	99 (181213)^‖^	90 (172497)^‖^	98	8
MMR-2	89 (172226)^‖^	97 (176845)^‖^	87 (167381)^‖^	93	6
DPT-booster	89 (172226)^‖^	97 (176845)^‖^	86 (164611)^‖^	93	7
	**Avg. %** **(Σ** *n * **)**	**Avg. %** **(Σ** *n * **)**	**Avg. %** **(Σ** *n * **)**	*P * ** value** ^#^ **2020 vs. 2018**	*P * ** value** ^#^ **2020 vs. 2019**
All-vaccines	93.5 (2,773,570)	90.7 (2,702,482)	79.6 (2,497,794)	<0.001^*∗*^	0.001^*∗*^

Avg.: average; BCG: Bacillus Calmette–Guérin; DPT: diphtheria, pertussis, and tetanus; MMR: measles, mumps, and rubella; OPV: oral polio vaccine; vs.: versus. ^†^*n*: number of vaccine doses administered. ^‡^Crude birth; 2018: 202711; 2019: 208792; 2020: 220057. ^§^Hexavalent vaccine: diphtheria, pertussis, tetanus (DPT); *Haemophilus influenzae* type b (Hib); hepatitis B vaccine (HBV); and inactivated polio vaccine (IPV). ^¶^Surviving infants; 2018: 198656; 2019: 204616; 2020: 215656. ^‖^Expected population of 1-2 years old; 2018: 194417; 2019: 182244; 2020: 192077. ^#^Kruskal–Wallis test. ^*∗*^*P* value <0.05 indicates significant differences.

**Table 3:** Sociodemographic characteristics of the caregivers/children and their association with the outcome measure “vaccination delay”^†^ (*N* = 568).

	%^‡^(*n*)	*P* value^§^
*Caregiver characteristics*		
Age (year) (median ± IQR)^¶^	30 ± 8	0.217^*∗*^^, ‖^
Gender/caregiver^††^		
Female	97.4% (553)	0.097
Male	2.6% (15)	
Monthly income^††^		
≤ 500 JD (∼700 $)	66.9% (380)	0.217
> 500 JD (∼700 $)	33.1% (188)	
Working in the medical field^††^		
Yes	12.1% (69)	0.51
No	87.9% (499)	
Marital status^††^		
Married	99.1% (563)	0.795
Others (divorced or widow(er))	0.9% (5)	
Relationship to the child^††^		
Mother	95.6% (543)	0.288
Father or others (grandmother, aunt, or uncle)	4.4% (25)	
Medical insurance^††^		
Insured	83.3% (473)	0.65
Not insured	16.7% (95)	
Self-described health status^††^		
Excellent, very good, or good	97.9% (556)	0.5
Weak or very weak	2.1% (12)	
Level of education^††^		
B.Sc. or higher	57.2% (325)	0.26
College or less	42.8% (243)	
Employment status^††^		
Full time or part time	29.0% (165)	0.311
Unemployed	71.0% (403)	
Number of children^††^		
≤3	72.2% (410)	0.066
>3	27.8% (158)	
Site of vaccination^††^		
Public health center only	89.3% (507)	0.434
Private clinic only	1% (6)	
Public health center and private clinic	0.9% (5)	
UNRWA	8.8% (50)	

*Child characteristics*		
Age (month)^††^		
<12	46.1% (262)	<0.001^*∗*^
≥12	53.9% (306)	
Gender^††^		
Female	46.1% (262)	0.145
Male	53.9% (306)	
Health status^††^		
Excellent, very good, or good	94.7% (538)	0.017^*∗*^
Weak or very weak	5.3% (30)	
Presence of chronic diseases^††^		
Yes	20.6% (117)	<0.001^*∗*^
No	79.4% (451)	

IQR: interquartile range; JD: Jordanian dinar. ^†^Delayed vaccination is defined as vaccination that was administered after one month or more of the recommended vaccination age besides those that were delayed and had not yet been administered by the time of the interview. ^‡^Valid percent (*N* = 568).^§,^^*∗*^*P* value <0.05 indicates a significant association of the covariate with the outcome measure of “vaccination delay.” ^¶^Eta test. ^‖^Eta coefficient: 0.2–0.39: weak association; 0.4–0.69: medium association; 0.7–1.0: strong association. ^††^Pearson's chi-square test.

**Table 4:** Beliefs of caregivers about childhood vaccination and adherence to childhood vaccination schedule during the COVID-19 pandemic (*N* = 568).

Beliefs	%^†^(*n*)	*P* value^‡^
Adherence to child vaccination schedule is essential^§^	93.1% (529)	<0.001^*∗*^
Vaccinations are important for child health^§^	90.5% (514)	0.620
There is a risk of acquiring COVID-19 in health centers^§^	71.5% (406)	0.453

^†^Valid percent (*N* = 568).^‡,^^*∗*^*P* value <0.05 indicates a significant association of the covariate with the outcome measure “vaccination delay.” ^§^Pearson's chi-square test.

**Table 5:** Practices of the caregivers who delayed child vaccination during COVID-19 pandemic (*n* = 142).

%^†^(*n*)	
*Duration of the delay*	
1 month	40.8% (58)
2 months	26.8% (38)
3 months	9.2% (13)
4 months or more	23.2% (33)

*Frequency of vaccination delay by age category*	
First-month vaccine (BCG)	18.3% (26)
Second-month vaccine (hexavalent-1^‡^; rotavirus-1)	13.4% (19)
Third-month vaccine (hexavalent-2^‡^; OPV-1; rotavirus-2)	19.7% (28)
Fourth-month vaccine (hexavalent-3^‡^; OPV-2; rotavirus-3)	20.4% (29)
Ninth-month vaccine (measles vaccine; OPV-3)	19.0% (27)
Twelfth-month vaccine (MMR-1)	19.0% (27)
Eighteenth-month vaccine (OPV-booster; DPT-booster; MMR-2)	33.1% (47)

*Time interval during which the vaccine was delayed*	
1 January 2020 (corona virus outbreak) to 29 January 2020	2.1% (3)
30 January 2020 (public health emergency of international concern) to 1 March 2020	0.7% (1)
2 March 2020 (first confirmed case of COVID-19 in Jordan) to 20 March 2020	0.7% (1)
21 March 2020 to 21 April 2020 (lockdown)	42.3% (60)
22 April 2020 to 30 May 2020 (lockdown, partially relaxed)	6.3% (9)
After 30 May 2020 (smart lockdown, first and second waves)	52.5% (74)

*Reasons for the delay*	
Lockdown	42.3% (60)
Sick child at the vaccine due date	31.7% (45)
Regional lockdown/health center closure due to COVID-19	7.7% (11)
Lack of time	4.9% (7)
Traveling	2.1% (3)
Forgetting	2.1% (3)
Physician advice to postpone	0.7% (1)

BCG: Bacillus Calmette–Guérin; DPT: diphtheria, pertussis, and tetanus; MMR: measles, mumps, and rubella; OPV: oral polio vaccine. ^†^Valid percent of those who delayed a child vaccine (*n* = 142). ^‡^Hexavalent vaccine: diphtheria, pertussis, tetanus (DPT); *Haemophilus influenzae* type b (Hib); hepatitis B vaccine (HBV); and inactivated polio vaccine (IPV).

**Table 6:** Predictors of vaccination delay during the COVID-19 pandemic in Jordan according to multiple logistic regression model.

Covariate	B	SE	*P* value	OR	95% CI
Age of the caregiver (years)	0.023	0.017	0.186	1.02	0.99–1.06
Age of the child (months)	−1.720	0.247	< 0.001^*∗*^	0.18	0.11–0.29
<12					
≥12^†^					
Child's chronic diseases	−0.628	0.253	0.013^*∗*^	0.53	0.33–0.88
No					
Yes^†^					
Child health status	0.565	0.441	0.201	1.76	0.74–4.18
Weak or very weak					
Excellent, very good, or good^†^					
Caregivers' belief in the importance of adhering to child vaccination schedule	1.418	0.365	<0.001^*∗*^	4.13	2.02–8.44
No					
Yes^†^					
**Constant**	**−0.057**	**0.989**	**0.954**	**0.95**	

OR: odds ratio. ^†^Reference category. ^*∗*^Significant at *P* value <0.05.

## Data Availability

Data are available upon request.
